# Antimicrobial Peptidomimetics Prevent the Development of Resistance against Gentamicin and Ciprofloxacin in Staphylococcus and Pseudomonas Bacteria

**DOI:** 10.3390/ijms241914966

**Published:** 2023-10-06

**Authors:** Katrina Browne, Rajesh Kuppusamy, William R. Walsh, David StC Black, Mark D. P. Willcox, Naresh Kumar, Renxun Chen

**Affiliations:** 1 School of Chemistry, University of New South Wales (UNSW) Sydney, Sydney 2052, Australia; 2 Surgical and Orthopaedic Research Laboratories (SORL), Prince of Wales Clinical School, Prince of Wales Hospital, University of New South Wales (UNSW), Randwick 2031, Australia; 3 School of Optometry and Vision Science, University of New South Wales (UNSW) Sydney, Sydney 2052, Australia

**Keywords:** antibiotic resistance, antibiotics, antimicrobial peptides, peptidomimetics

## Abstract

Bacteria readily acquire resistance to traditional antibiotics, resulting in pan-resistant strains with no available treatment. Antimicrobial resistance is a global challenge and without the development of effective antimicrobials, the foundation of modern medicine is at risk. Combination therapies such as antibiotic–antibiotic and antibiotic–adjuvant combinations are strategies used to combat antibiotic resistance. Current research focuses on antimicrobial peptidomimetics as adjuvant compounds, due to their promising activity against antibiotic-resistant bacteria. Here, for the first time we demonstrate that antibiotic–peptidomimetic combinations mitigate the development of antibiotic resistance in *Staphylococcus aureus* and *Pseudomonas aeruginosa*. When ciprofloxacin and gentamicin were passaged individually at sub-inhibitory concentrations for 10 days, the minimum inhibitory concentrations (MICs) increased up to 32-fold and 128-fold for *S. aureus* and *P. aeruginosa*, respectively. In contrast, when antibiotics were passaged in combination with peptidomimetics (Melimine, Mel4, RK758), the MICs of both antibiotics and peptidomimetics remained constant, indicating these combinations were able to mitigate the development of antibiotic-resistance. Furthermore, antibiotic–peptidomimetic combinations demonstrated synergistic activity against both Gram-positive and Gram-negative bacteria, reducing the concentration needed for bactericidal activity. This has significant potential clinical applications—including preventing the spread of antibiotic-resistant strains in hospitals and communities, reviving ineffective antibiotics, and lowering the toxicity of antimicrobial chemotherapy.

## 1. Introduction

Antimicrobial resistance is one of the grand challenges of the 21st century. Bacteria are developing resistance towards antibiotics at an alarming rate [1,2]. The use of antibiotics to prevent and treat infection is intrinsically linked to modern medicine [3]. Without reliable antibiotic therapy, the infection risk of common procedures, such as surgery, cancer chemotherapy and childbirth, increases dramatically [4,5]. This would result in significant morbidity and mortality for patients, and a fiscal burden for the healthcare industry [6]. If the number of antibiotic-resistant infections continues to rise, by 2050 it is predicted there will be 10 million deaths per year, with a financial burden of USD 100 trillion [7]. The current approaches to prevent the spread of antibiotic-resistant bacteria centre around antimicrobial stewardship and correct hygiene procedures [8]. However, these measures are not completely effective and antibiotic-resistant microbes continue to emerge [7,9].

The mechanisms of antibiotic resistance are complex, but can be summarised into four key categories [10]. Bacteria can modify the drug target, preventing the action of the antibiotic. Secondly, bacteria can increase the expression of efflux pumps to flux antibiotics from inside the cell. Thirdly, bacteria can produce specialised enzymes to degrade antibiotics. Finally, bacteria can modify the bacterial membrane and receptors to stop the entry of the antibiotic into the cell. These mechanisms of resistance all revolve around inhibiting antibiotics from acting inside the cell. Thus, there remains a need for novel-acting antibiotics that address the drawbacks of current antibiotics.

**Figure 1:**
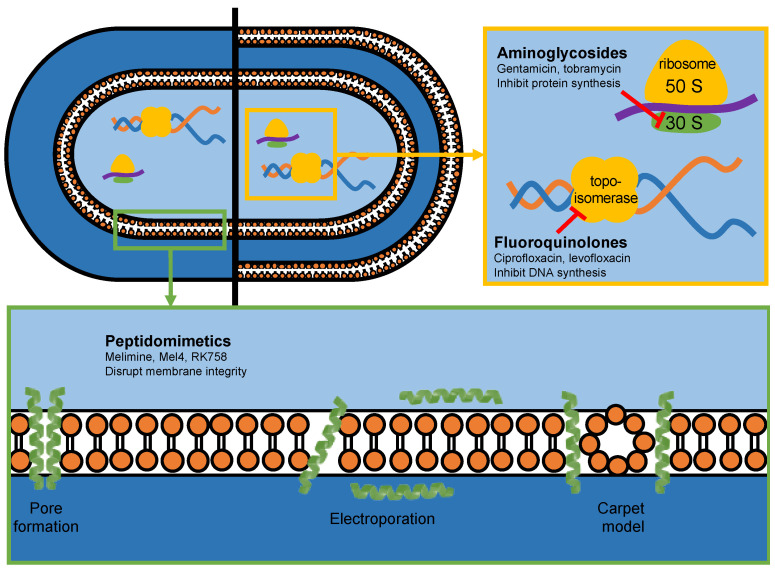
The antibacterial mechanism of action of antibiotics (yellow box) and peptidomimetics (green box) used in this study.

Antimicrobial peptides are an emerging class of antibiotics that show great clinical promise [11]. Peptidomimetics are designed to mimic natural peptides, with the ability to alter their chemical structure to improve activity, stability and biocompatibility [12]. Pelay-Gimeno et al. established a classification system for peptidomimetics, based on the degree of modifications to the natural peptide structure [12]. Melimine and Mel4 are modifications of the natural antimicrobial peptides melittin and protamine, and are thus categorised as class A peptidomimetics. RK758 is a synthetic compound designed to mimic the mechanistic properties of antimicrobial peptides and is categorised as a class D peptidomimetic. For simplicity, Melimine, Mel4 and RK758 are referred to as peptidomimetics in this study. These peptidomimetics have shown potent antibacterial activity against antibiotic-resistant strains, low toxicity to mammalian cells and antibiofilm activity [13,14,15]. Peptidomimetics have a distinct mechanism of action compared to traditional antibiotics (Figure 1).

Due to their amphipathic nature, peptidomimetics are thought to disrupt bacterial cell membrane integrity, resulting in rapid cell lysis and death [16,17,18,19]. As peptidomimetics do not need to be internalised by the cell for activity, they provide an effective strategy to overcome several antibiotic resistance mechanisms [15,20]. Furthermore, bacteria have a lower propensity to developing resistance towards peptidomimetics, due to their action on the evolutionarily conserved cell membrane [21,22].

Many researchers have suggested that peptidomimetics are a solution to the antibiotic resistance crisis [23,24,25]. However, antibiotics are the gold standard of care, and will be for the foreseeable future. Adjunct therapy with peptidomimetics would offer a novel solution for treating the most difficult infections, where current antibiotic therapy is ineffective. From a clinical perspective, antibiotic-resistant infections on medical devices, such as prosthetic implants, are the most difficult to treat [26]. Briefly, 3D-printed biomaterials offer an increased surface area for these bacteria to attach and form biofilms. Once a biofilm is established, the infected material requires a two-stage revision surgery, and aggressive treatment with antibiotics, which would be ineffective in the case of antibiotic-resistant strains. The failure rates of revision surgeries are as high as 34% [27]. Thus, inhibiting these types of infections is an essential need in the use of medical devices, where combination therapy strategies may offer a novel solution to this crisis.

We believe that the combination therapy of conventional antibiotics and peptidomimetics is a promising strategy to limit the development of antibiotic resistance and prolong the activity of essential antibiotics [28,29,30,31]. This study assesses how bacteria respond to antimicrobial combinations in vitro, and the potential clinical significance of adjuvant peptidomimetic therapy.

## 2. Results

### 2.1. Antimicrobial Activity against Clinical Bacterial Isolates

Two antibiotics (gentamicin and ciprofloxacin) and three peptidomimetics (Melimine, Mel4 and RK758) were tested for their minimum inhibitory concentration (MIC) against a panel of Gram-positive and Gram-negative bacteria (Table 1). We included an antibiotic-resistant strain for each species to assess the activity of peptidomimetics. The *S. aureus* 31 strain is a multi-drug resistant isolate, resistant to antibiotics including ciprofloxacin, ceftazidime, azithromycin and polymyxin [32]. Resistance towards ciprofloxacin was confirmed in our study. The *E. coli* NCTC 13846 is a colistin-resistant isolate which confers resistance to other membrane-active compounds, including polymyxin antibiotics [33]. This strain was completely resistant towards ciprofloxacin. The *P. aeruginosa* 123 strain is a colistin-resistant isolate which confers resistance to other membrane-active compounds, including polymyxin antibiotics [34]. This strain showed no altered activity for the antimicrobials tested. All three peptidomimetics showed activity against antibiotic-resistant strains (*S. aureus* 31, *E. coli* NCTC 13846 and *P. aeruginosa* 123), with no evidence of increased MICs compared to those of the sensitive strains. As expected, the MIC for Gram-negative strains trended higher than did that for Gram-positive strains, especially for peptidomimetics. Of the peptidomimetics, the smallest-molecular-weight compound, RK758, showed the lowest MIC (3–100 µM), compared to that of Melimine and Mel4, for all strains tested.

### 2.2. In Vitro Interactions between Antimicrobial Combinations

**Figure 2:**
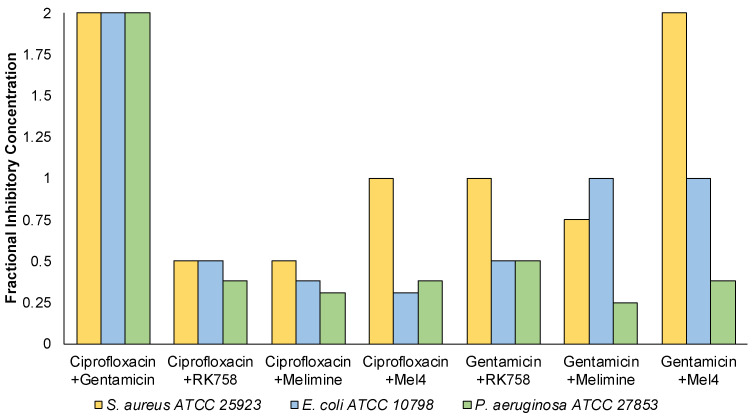
Summary of the fractional inhibitory concentration of antimicrobial combinations against *Staphylococcus aureus, Escherichia coli* and *Pseudomonas aeruginosa*. *n* = 1.

A checkerboard assay was used to quantify antibacterial activity when two compounds were used in combination against representative strains of *S. aureus*, *E. coli* and *P. aeruginosa*. This assay allowed the determination of individual MIC values of antimicrobials used in combination. The individual MIC values were used to calculate the fractional inhibitory concentration (FIC) and assess combination activity. When the antibiotics ciprofloxacin and gentamicin were used in combination, there was no effect (FIC = 2) on their individual MIC (Figure 2). In contrast, when ciprofloxacin was used in combination with peptidomimetics, the FIC ranged between 1–0.38. There was synergistic activity (FIC ≤ 0.5) for all strains for the ciprofloxacin–RK758 and ciprofloxacin–Melimine combinations. For Gram-negative bacteria, the ciprofloxacin–Mel4 combinations had synergistic activity against *E. coli* (FIC = 0.31) and *P. aeruginosa* (FIC = 0.38). Similarly, Gentamicin–RK758 combinations showed synergistic activity for both strains (FIC = 0.5). Against *P. aeruginosa,* synergistic activity was observed for gentamicin–Melimine (FIC = 0.25) and gentamicin–Mel4 (FIC = 0.38). There was no antagonism (FIC ≥ 4) for any combination.

**Figure 3:**
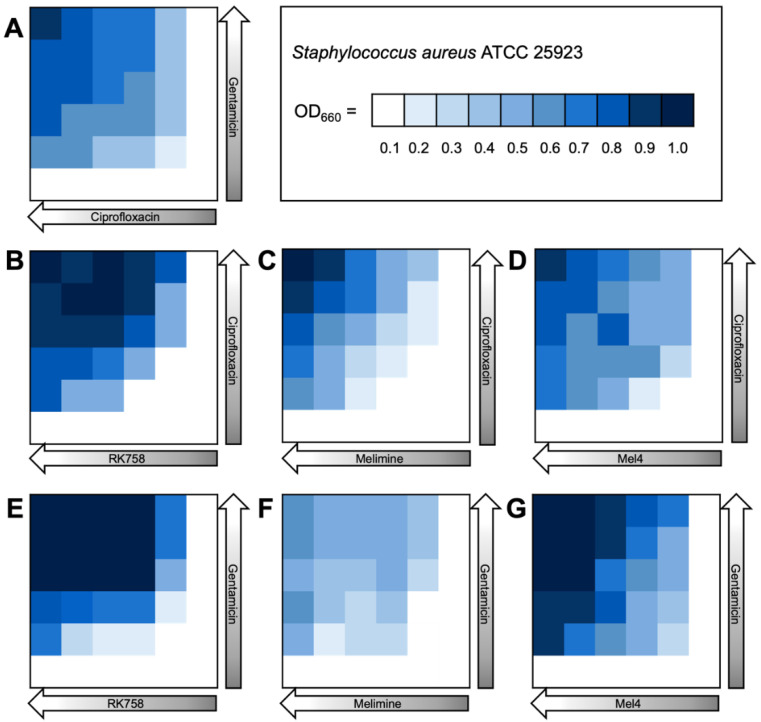
Bacterial density maps of combination therapies in *Staphylococcus aureus* ATCC 22923. OD660 = optical density at 660 nm. (**A**) Gentamicin–ciprofloxacin, (**B**) ciprofloxacin–RK758, (**C**) ciprofloxacin–Melimine, (**D**) ciprofloxacin–Mel4, (**E**) gentamicin–RK758, (**F**) gentamicin–Melimine (**G**) and gentamicin–Mel4.

**Figure 4:**
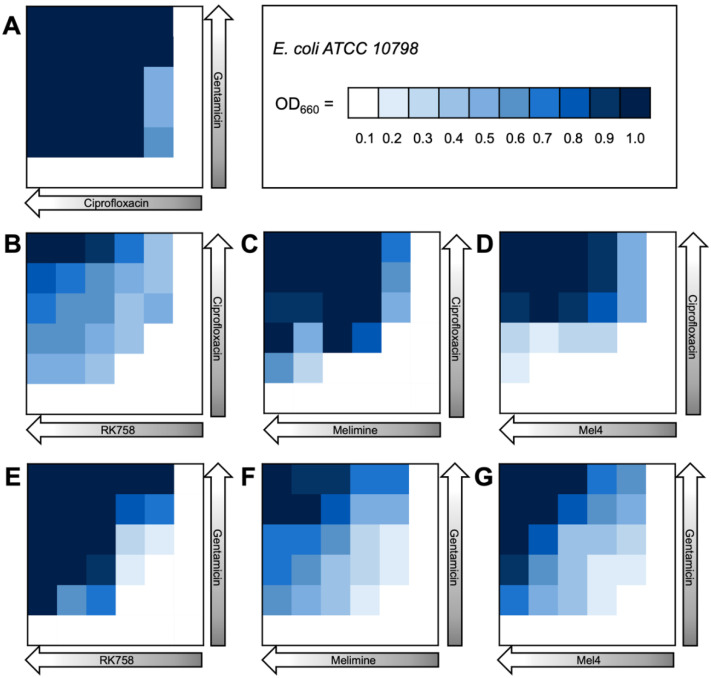
Bacterial density maps of combination therapies in *Escherichia coli* ATCC 10798. OD660 = optical density at 660 nm. (**A**) Gentamicin–ciprofloxacin (**B**) ciprofloxacin–RK758, (**C**) ciprofloxacin–Melimine, (**D**) ciprofloxacin–Mel4, (**E**) gentamicin–RK758, (**F**) gentamicin–Melimine and (**G**) gentamicin–Mel4.

**Figure 5:**
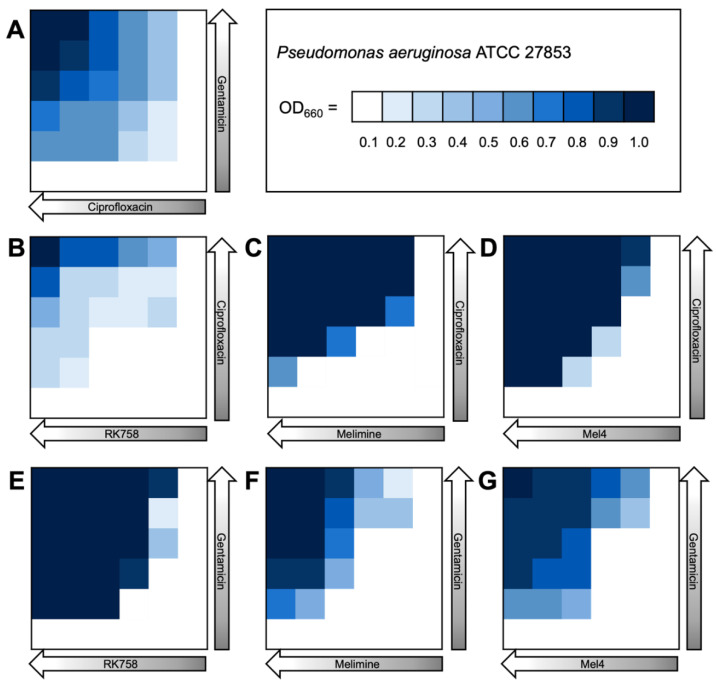
Bacterial density maps of combination therapies in *Pseudomonas aeruginosa* ATCC 27853. OD660 = optical density at 660 nm. (**A**) Gentamicin–ciprofloxacin, (**B**) ciprofloxacin–RK758, (**C**) ciprofloxacin–Melimine (**D**), ciprofloxacin–Mel4, (**E**) gentamicin–RK758, (**F**) gentamicin–Melimine and (**G**) gentamicin–Mel4.

The bacterial density maps give more insight into the interactions between antibiotics and peptidomimetics. For *S. aureus* the FIC value for the gentamicin–Melimine combination was 0.75, where gentamicin could be used at 0.25× the MIC and Melimine could be used at 0.5× the MIC (Figure 3F). Contrastingly, for *E. coli* the FIC value for ciprofloxacin–Melimine was 0.38, where ciprofloxacin could be used at 0.5× the MIC and Melimine could be used at 0.125× the MIC (Figure 4C). Similarly, Mel4 could be used at 0.06× the MIC when combined with ciprofloxacin at 0.5× the MIC against *E. coli* (Figure 4D). For *P. aeruginosa*, ciprofloxacin could be used at 0.25× the MIC when combined with either RK758 at 0.125× (Figure 5B) or Melimine at 0.06× the MIC (Figure 5C). Ciprofloxacin could be used at 0.125× the MIC when used in combination with Mel4 at 0.25× the MIC (Figure 5D). Gentamicin could be used at 0.125× the MIC when combined with Melimine at 0.125× (Figure 5F) and Mel4 at 0.25× the MIC (Figure 5G).

### 2.3. Repeated Passage of Bacteria in Sub-MIC Antimicrobials

Two strains of bacteria (*S. aureus ATCC 25923* and *P. aeruginosa ATCC 27853*) were passaged in sub-inhibitory concentrations of antimicrobials for 10 days. For *S. aureus*, the MIC for gentamicin or ciprofloxacin increased 128-fold throughout the 10-day period (Table 2, Supplementary Table S1). In contrast, when ciprofloxacin or gentamicin was used in combination with either one of the three peptidomimetics, the MIC of both antibiotics at Day 10 remained the same as that at Day 0 (Table 2). When ciprofloxacin and gentamicin were used in combination, the MIC increased two-fold for each antibiotic. There was no change in the MIC for RK758, Melimine and Mel4 after the 10-day passage. Even when the assay was extended to 30 days, the MIC for the peptidomimetics remained the same as that at Day 0 (Supplementary Table S1). For *P. aeruginosa*, the MIC for gentamicin and ciprofloxacin increased 32-fold and 16-fold throughout the 10-day period, respectively (Table 3, Supplementary Table S2). Like what was observed for *S. aureus*, when ciprofloxacin or gentamicin was used in combination with either one of the peptidomimetics, the MIC of both antibiotics remained the same as that in the 10-day period (Table 3). When ciprofloxacin and gentamicin were used in combination, the MIC increased four-fold for each antibiotic. Similarly, The MIC against *P. aeruginosa* for RK758, Melimine and Mel4 on their own remained the same as that at Day 0 after 30 consecutive passages (Supplementary Table S3).

### 2.4. Cross-Resistance MIC

Bacteria acquired resistance to ciprofloxacin and gentamicin when consecutively passaged for 10 days in sub-inhibitory concentrations (Table 2 and Table 3). These Day 10 antibiotic-resistant strains were used to determine the MIC of other related compounds, including tobramycin and levofloxacin in addition to the five previously tested antibiotic and peptidomimetics. For the *S. aureus* gentamicin-resistant bacteria, the MIC of tobramycin increased 32-fold (Table 4). There was no change in the MIC for the other antimicrobials tested (Table 4). For the *S. aureus* ciprofloxacin-resistant bacteria, the MIC of levofloxacin increased 16-fold (Table 4). There was no change in the MIC for the other antimicrobials tested (Table 4). For the *P. aeruginosa* gentamicin-resistant bacteria, the MIC of tobramycin increased 64-fold (Table 5). There was no change in the MIC for the other antimicrobials tested (Table 5). For the *P. aeruginosa* ciprofloxacin-resistant bacteria, the MIC of levofloxacin increased 128-fold (Table 5). There was no change in the MIC for the other antimicrobials tested (Table 5).

## 3. Discussion

This study assessed the potential for combination therapy to reduce resistance towards antibiotics. We found that bacteria did not acquire resistance to gentamicin or ciprofloxacin, when passaged in sub-inhibitory antibiotic–peptidomimetic combinations. These combination strategies have the potential to reduce the rate that antibiotic-resistant isolates develop, thus prolonging the effectiveness of currently available antibiotics. Previous research has suggested that peptidomimetics may protect against antibiotic resistance development, due to the unique action of peptidomimetics on the bacterial cell membrane [35,36]. To our knowledge, this is the first study to demonstrate that antibiotic–peptidomimetic combinations mitigated the development of antibiotic resistance.

Combination therapy is a common clinical strategy used to limit resistance towards cancer chemotherapy, as well as multidrug-resistant bacterial infections. Thus, we hypothesised that combination antibiotic–peptidomimetic therapy may reduce the rate that bacteria acquire resistance towards antibiotics. To test this hypothesis, bacteria were passaged over a 10-day period in sub-MIC antibiotic–peptidomimetic combinations. When ciprofloxacin and gentamicin were passaged individually at sub-inhibitory concentrations for 10 days, the minimum inhibitory concentrations (MICs) increased up to 32-fold and 128-fold for *S. aureus* and *P. aeruginosa*, respectively (Table 2 and Table 3). There was no change in the MIC after 10 days when both strains were in antibiotic–peptidomimetic combinations (Table 2 and Table 3). Our results demonstrate that combination antibiotic–peptidomimetic treatment completely mitigated the development of antibiotic resistance towards ciprofloxacin and gentamicin, for both *S. aureus* and *P. aeruginosa*.

While the precise molecular mechanisms for this are still unknown, we propose that peptidomimetics activate specific bacterial cell stress response pathways. It is known that when bacterial homeostasis is disrupted, stress response pathways are activated [37,38]. In response, bacteria slow cell cycle progression, reduce proliferation, alter the production of virulence factors and induce biofilm formation [39,40,41,42,43]. As the genetic instructions for bacterial cell membrane composition are evolutionarily conserved, large numbers of simultaneous mutations would be required for bacteria to re-design the structure of their cell membranes [44]. When antibiotics are combined with peptidomimetics that act on this cell membrane, there are multiple cell stress response pathways that are activated. The overall chance of resistant isolates developing with all the necessary mutations to overcome this is significantly reduced. This is demonstrated by the ciprofloxacin–gentamicin combination reducing the rate of resistance compared to that when monotherapies are used. However, after 10 days there were two-fold and four-fold increases in the MIC for *S. aureus* and *P. aeruginosa*, respectively (Table 2 and Table 3). This suggests a slower rate of resistance, but not complete protection.

Checkerboard assays showed that antibiotic–peptidomimetic combinations had synergistic activity in both Gram-positive and Gram-negative bacterial strains (Figure 2). Overall, the lowest FIC values were seen with ciprofloxacin in combination with peptidomimetics, particularly in Gram-negative bacteria. Surprisingly, the greatest activity was seen in *P. aeruginosa*, with all antibiotic–peptidomimetic combinations showing synergistic activity. As Gram-negative bacteria have a double-membrane structure, many antibiotics cannot pass through to reach their intracellular targets [45]. The checkerboard assays were conducted with *n* = 1, due to the number of combinations and number of strains used in this study. Further, the checkerboard assays were primarily used to inform us on the dosage of the antimicrobial combinations to be tested in the repeated passage experiments (Section 2.3). This limitation prevented any statistical analysis of the FIC results, and further replicates of the checkerboard assays would need to be carried out to confirm the preliminary determination of the synergistic/antagonistic effects shown here.

We propose the synergistic effect is due to the membrane-poration caused by peptidomimetics below the MIC, allowing the antibiotic to enter the cell, causing cell death. Previous studies showed synergistic activity against *S. aureus*, *E.coli* and *P. aeruginosa* when membrane-disrupting agents were used in combination with antibiotics [46,47,48]. This is further supported by the lower synergistic effect we saw for *S. aureus*, as these membranes are generally more permeable compared to the outer membrane of *P. aeruginosa*. As *P. aeruginosa* infections are often the most difficult to treat clinically, this strategy offers a solution to not only improve the efficacy of treatment, but also the potential to lower treatment doses and reduce toxicity [49,50].

The three peptidomimetics (Melimine, Mel4, and RK758) used in this study were chosen for their broad-spectrum antibacterial activity, including that against antibiotic-resistant clinical isolates (Table 1). Interestingly, Melimine and Mel4 showed no change in the MIC against polymyxin-resistant strains (*E. coli* NCTC 13846 and *P. aeruginosa* 123) [51], although they were previously hypothesised as having a similar mechanism of action to that of polymyxin antibiotics. When comparing the concentrations required for activity, both Melimine and Mel4 require significantly higher concentrations than do polymyxin antibiotics. Thus, while they may have overlapping targets, these compounds may act via an alternate mechanism against these polymyxin-resistant strains [52]. The smaller-molecular-weight compound, RK758, showed the greatest activity against these strains. While the mechanism of action for RK758 has not been elucidated, we previously hypothesised it has a distinct mechanism compared to that of polymyxin antibiotics that interact with negatively charged lipopolysaccharides [15].

These peptidomimetics have shown good biocompatibility, safety and efficacy in preclinical and in vivo studies including a Phase I/II human clinical trial [15,20,53,54,55]. It is their unique mechanism of action that has the potential to lower the rate of antibiotic resistance development. Due to their indiscriminate action on the bacterial cell membrane, rather than on specific intracellular targets, bacteria would need to redesign the composition of the cell envelope to develop resistance [44]. This envelope is evolutionarily conserved, and would require multiple, simultaneous genetic mutations for bacteria to acquire resistance [44]. Indeed, both *S. aureus* and *P. aeruginosa* failed to acquire resistance towards peptidomimetics, throughout a 30-day repeated passage (Table 2 and Table 3).

In this study, bacteria rapidly acquired resistance towards mono-antibiotic therapies. Previous studies suggest that these bacteria can also develop cross-resistance between unrelated drug classes [56]. To assess if these resistance mutations conferred cross-resistance to other antibiotics, we determined the MIC of several antimicrobials against the Day 10 antibiotic-resistant isolates and compared them to that of the Day 0 controls. After 10 days, there was cross-resistance seen between antibiotics of the same class (ciprofloxacin and levofloxacin, and gentamicin and tobramycin) (Table 4 and Table 5). There was no cross-resistance seen between distinct antibiotic classes (aminoglycosides and fluoroquinolones). The data suggest that these antibiotic resistance-conferring mutations are specific to the drug target of antibiotics, and as aminoglycosides and fluoroquinolone antibiotics act on distinct biological targets there was no effect on activity for tobramycin and levofloxacin. These findings are in agreement with those of a a study by Zheng et al. where no cross-resistance was found between ciprofloxacin- and gentamicin-resistant isolates [57]. As expected, the Day 10 antibiotic-resistant isolates showed no change in the MIC for peptidomimetics due to their distinct mechanism compared to that of these antibiotics.

Overall, the results represent compelling evidence that the peptidomimetics protected bacteria from developing resistance towards antibiotics. This is of huge significance, both for the clinic and the community in reducing the rate of antibiotic-resistant infections [58]. As the spread of antibiotic resistance continues to rise, the personal and financial burden increases for patients [6]. In addition to the protective nature of adjunct peptidomimetic therapy, this combination may be able to improve the activity of antibiotics against drug-resistant bacterial strains. Previous studies have shown the potential for combination therapies of known drugs and additives to work in synergy with frontline antibiotics, including reverting resistance to colistin [58,59,60]. Future work should assess the ability of peptidomimetics treatment to re-sensitise antibiotics to resistant strains. This would vastly improve the arsenal of effective antibiotics available to treat bacterial infections. However, for this strategy to be effective, the mechanism of resistance is important, as a mutation in the drug target would still preclude antibiotic activity.

While this study is the first to demonstrate the protective nature of antibiotic–peptidomimetic combinations, it is important to consider the limitations when translating these findings to the clinic. The conditions of in vitro experiments are not true representations of those in in vivo environments [61]. Outside factors in clinical scenarios, such as patient-specific factors and environmental contamination, would need to be considered [62]. While we show that both *S. aureus* and *P. aeruginosa* rapidly acquired resistance towards commercial antibiotics, the testing of specific gene mutations was outside the scope of this study. Additionally, while no resistance was seen towards peptidomimetics in the 30-day period, resistance towards antimicrobials is an evolutionary consequence of their use [63]. Thus, the use of peptidomimetics must be considered in line with antimicrobial stewardship guidelines.

This study is pivotal, and the first to demonstrate the protective nature of adjunct peptidomimetic therapy. If used appropriately, this study shows the promise of peptidomimetics as effective antimicrobial adjuvants to reduce the rate of antibiotic resistance development. With the decline in the development of effective antimicrobials, peptidomimetics offer a novel strategy to prolong the effectiveness of current antibiotics.

## 4. Materials and Methods

### 4.1. Bacterial Strains

Three species of bacteria were chosen (*Staphylococcus aureus*, *Escherichia coli*, and *Pseudomonas aeruginosa*) to broadly represent clinically relevant pathogens. *S. aureus* ATCC 25923, *E. coli* ATCC 10798 (K12) and *P. aeruginosa* ATCC 27853 were purchased from American Type Culture Collection (ATCC). *S. aureus* 38 is a clinical strain isolated from a corneal ulcer [64]. *S. aureus* 31 is a multidrug (ciprofloxacin, ceftazidime, azithromycin and polymyxin)-resistant clinical isolate from a corneal infiltrative event [32]. *E. coli* NCTC 13846 is a colistin-resistant, *mcr-1* positive strain purchased from National Collection of Type Cultures (NCTC) [33]. *P. aeruginosa* O1 is a clinical strain isolated from a patient burn wound [65]. *P. aeruginosa* 123 is a polymyxin B-resistant strain isolated from a corneal ulcer [34].

### 4.2. Antimicrobial Agents

The antibiotics gentamicin, tobramycin, ciprofloxacin and levofloxacin were purchased from Sigma Aldrich (Burlington, MA, USA). Melimine and Mel4 were purchased from AusPep Peptide Company (Tullamarine, VIC, Australia). The purity of the purchased peptides was ≥90%. RK758 was synthesised as previously described [15], according to the patents (WO2018081869A1 and Australian Provisional Patent Application No. 2021902457).

### 4.3. Bacterial Culture and Conditions

For all strains tested, a single bacterial colony was incubated in cation-adjusted Mueller-Hinton Broth (MHB-II; Oxoid, Basingstoke, UK) and cultured for 18 h at 37 °C. Bacterial solutions were then centrifuged at 5000× *g*, and the supernatant was discarded and refreshed with MHB-II. A spectrophotometer was used to quantify the optical density at 660 nm (OD_660_) of the bacterial solution. Using this value, the bacterial solution was adjusted to ~10^5^ CFU*mL^−1^.

### 4.4. Minimum Inhibitory Concentration

The MIC was determined using the microtiter broth dilution method [66]. Briefly, antimicrobial compounds were prepared in MHB-II and serially diluted (1:2) on a 96-well polystyrene microplate (COSTAR Corning Incorporated, New York, NY, USA). Each compound was tested in duplicate. After serial dilutions, each well contained 100 µL of antimicrobial solution. This was followed by the addition of 100 uL of the prepared bacterial solution (~10^5^ CFU*mL^−1^) to each well. Negative control wells were plated with 200 µL of MHB-II only. Positive control wells were prepared without antimicrobial compounds (200 µL). The plate was sealed with a protective film (SealPlate^®^, Sigma Aldrich, St. Louis, MO, USA) to prevent evaporation and cross-contamination. The plate was then incubated at 37 °C for 18 h. Following incubation, the OD_660_ was measured using a spectrophotometer. The MIC was determined as the lowest concentration that reduced bacterial growth by ≥90% compared to that of positive controls.

### 4.5. Fractional Inhibitory Concentration

**Figure 6:**
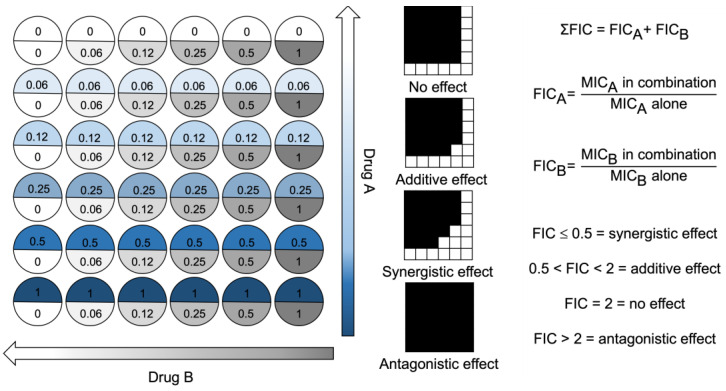
Checkerboard assay plate layout for two drugs used in combination. Well values represent ×MIC. Drug concentration arrows represent decreasing drug concentrations as the colour fades to zero. FIC = fractional inhibitory concentration. MIC = minimum inhibitory concentration.

The FIC was determined using a checkerboard microtiter broth dilution method (Figure 6). Briefly, two antimicrobial compounds were serially diluted in a perpendicular manner, corresponding to a gradient of combination concentrations. After serial dilutions, each well contained 100 µL of antimicrobial solution. The prepared bacterial solution (~10^5^ CFU*mL^−1^) was added in equal volume (100 µL) to each well. Negative control wells were plated with 200 µL of MHB-II only. Positive control wells were prepared without antimicrobial compounds (200 µL). The plate was sealed with a protective film (SealPlate^®^, Sigma Aldrich, St. Louis, MO, USA) to prevent evaporation and cross-contamination. The plate was then incubated at 37 °C for 18 h. Following incubation, the OD_660_ was measured using a spectrophotometer. The ∑ FIC value is calculated via the addition of FIC_Drug A_ + FIC_Drug B_. The FIC was used to assess the effect of using two antimicrobial compounds in combination, at various concentration gradients. The plate layout is illustrated in Figure 2, detailing the concentration in relation to the MIC of compounds A and B.

### 4.6. Repeated Passage of Antimicrobial Monotherapies

*S. aureus* ATCC 25923 and *P. aeruginosa* ATCC 27853 were passaged daily in antimicrobial compounds at 0.5× the MIC. In sterile Eppendorf tubes, 10 µL of bacterial overnight culture was added to 990 µL of MHB II, per the literature protocols [67]. Samples were incubated for 24 h at 37 °C, and each day a 10 µL aliquot was re-passaged in fresh media at 0.5× the MIC of the antimicrobial compound. Briefly, 20 µL from each sample was plated onto tryptic soy agar daily to detect the cross-contamination of bacteria. The MIC was tested daily for each compound, and if the MIC had increased, the concentration of the antimicrobial in solution was increased to remain at 0.5× the MIC. Day 0 and Day 10 MIC values were compared to determine how bacteria responded to prolonged, sub-inhibitory concentrations of antimicrobials. If no change in the MIC was observed, bacteria were passaged for a total of 30 days. Daily, bacterial cultures were stored in 25% (*v*/*v*) glycerol at −80 °C.

### 4.7. 10-Day Repeated Passage of Combination Antimicrobial Therapies

*S. aureus* ATCC 25923 and *P. aeruginosa* ATCC 27853 were passaged daily in the presence of two antimicrobial compounds. As combination antimicrobial therapy can alter the individual MIC value of each compound, the FIC data were used to determine the concentration of antimicrobial compounds, to the equivalent of 0.5× the MIC when used in combination. In sterile Eppendorf tubes, 10 µL of bacterial overnight culture was added to 990 µL of MHB II, per the literature protocols [67]. Samples were incubated for 24 h at 37 °C, and each day a 10 µL aliquot was re-passaged in fresh media at 0.5× the MIC for each of the antimicrobial compounds. Briefly, 20 µL from each sample was plated onto tryptic soy agar daily to detect the cross-contamination of bacteria. The FIC was tested daily for each combination, and if the MIC of an antimicrobial compound had increased, the concentration of the antimicrobial in solution was increased to remain at 0.5× the MIC. Day 0 and Day 10 FIC values were compared to determine how bacteria responded to prolonged, sub-inhibitory concentrations of antimicrobial combinations. Daily bacterial cultures were stored in 25% (*v*/*v*) glycerol at −80 °C.

## Figures and Tables

**Table 1:** Minimum inhibitory concentration (MIC) of antimicrobial compounds against *Staphylococcus aureus*, *Escherichia coli* and *Pseudomonas aeruginosa*. * Indicates antibiotic-resistant strains.

Bacterial Strains		MIC (µM)				
		Antibiotics		Peptidomimetics		
		Gentamicin	Ciprofloxacin	Melimine	Mel4	RK758
Gram-positive	*S. aureus* ATCC 25923	1	1	25	100	8
	*S. aureus* 31 *	1	4	3	6	3
	*S. aureus* 38	0.5	0.5	4	6	3
Gram- negative	*E. coli* ATCC 10798 (K12)	1	0.5	20	75	16
	*E. coli* NCTC 13846 *	4	>250	8	16	8
	*P. aeruginosa* ATCC 27853	4	0.25	250	500	100
	*P. aeruginosa* O1	2	2	250	250	64
	P. aeruginosa 123 *	4	0.25	250	500	16

**Table 2:** Resistance evolution in *Staphylococcus aureus* ATCC 25923.

Antimicrobial Treatment		Minimum Inhibitory Concentration (µM)			
		Day 0		Day 10	
Gentamicin		1		32	
Ciprofloxacin		1		16	
Melimine		25		25	
Mel4		100		100	
RK758		8		8	
Gentamicin	Ciprofloxacin	1	1	2	2
Gentamicin	Melimine	0.25	12	0.25	12
Gentamicin	Mel4	1	100	1	100
Gentamicin	RK758	0.5	4	0.5	4
Ciprofloxacin	Melimine	0.25	6	0.25	6
Ciprofloxacin	Mel4	1	50	1	50
Ciprofloxacin	RK758	0.25	2	0.25	2

**Table 3:** Resistance evolution in *Pseudomonas aeruginosa* ATCC 27853.

Antimicrobial Treatment		Minimum Inhibitory Concentration (µM)			
		Day 0		Day 10	
Gentamicin		4		512	
Ciprofloxacin		0.25		32	
Melimine		250		250	
Mel4		500		500	
RK758		100		100	
Gentamicin	Ciprofloxacin	4	0.25	16	1
Gentamicin	Melimine	0.12	50	0.12	50
Gentamicin	Mel4	4	500	4	500
Gentamicin	RK758	4	50	4	50
Ciprofloxacin	Melimine	0.03	50	0.03	50
Ciprofloxacin	Mel4	0.06	125	0.06	125
Ciprofloxacin	RK758	0.03	25	0.03	25

**Table 4:** Antibiotic cross-resistance in *Staphylococcus aureus ATCC* 25923.

	Minimum Inhibitory Concentration (µM)		
Antimicrobial Treatment	Day 10 Control	Day 10 Ciprofloxacin-Resistant	Day 10 Gentamicin-Resistant
Gentamicin	1	1	32
Tobramycin	1	1	32
Ciprofloxacin	1	16	1
Levofloxacin	0.5	8	0.5
Melimine	3	3	3
Mel4	25	25	25
RK758	8	8	8

**Table 5:** Antibiotic cross-resistance in *Pseudomonas aeruginosa* 27853.

	Minimum Inhibitory Concentration (µM)		
Antimicrobial Treatment	Day 10 Control	Day 10 Ciprofloxacin-Resistant	Day 10 Gentamicin-Resistant
Gentamicin	4	4	256
Tobramycin	4	4	256
Ciprofloxacin	0.25	32	0.25
Levofloxacin	0.5	64	0.5
Melimine	400	400	400
Mel4	400	400	400
RK758	62	62	62

## Data Availability

The data presented in this study are available on request from the corresponding authors.

## Supplementary Materials

The following supporting information can be downloaded at https://www.mdpi.com/article/10.3390/ijms241914966/s1.
