# Supplementary material for: Antimicrobial Peptidomimetics Prevent the Development of Resistance against Gentamicin and Ciprofloxacin in Staphylococcus and Pseudomonas Bacteria
Source: Int J Mol Sci. 2023 Oct 6;24(19):14966. doi: 10.3390/ijms241914966 (PMC10573972; doi:10.3390/ijms241914966)
Supplement: Supplementary file 1 [file ijms-24-14966-s001.zip › ijms-2602567-supplementary.pdf]

Raw data. The summary data is presented in the manuscript for changes in minimum inhibitory concentrations (MICs) across the 10-day (Table S1, Table S2) or 10-day consecutive passage (Table S3). The following tables demonstrate the daily MICs for antimicrobial compounds and fractional MICs for combination therapy. For interpretation of combination study (Table S2 and Table S3) antimicrobials have been colour-coded where yellow = gentamicin, blue = ciprofloxacin, green = Melimine, orange = Mel4, purple = RK58.

Table S1. MIC (µM) determination over a 10-day consecutive passage of *Staphylococcus aureus* 25923 in antimicrobial compounds and antimicrobial combinations. FIC = fractional inhibitory concentration. Gen = gentamicin. Cip = ciprofloxacin. Mel = Melimine.

			Combination MIC																						
	Day		0		FIC	1		2		3		4		5		6		7		8		9		10	
S. aureus 25923	Gen		1		-	1		4		4		4		4		8		16		32		32		32	
	Cip		1		-	1		2		4		8		8		8		8		8		8		16	
	Mel		25		-	25		25		25		25		25		25		25		25		25		25	
	Mel4		100		-	100		100		100		100		100		100		100		100		100		100	
	RK758		8		-	8		8		8		8		8		8		8		8		8		8	
	Gen	Cip	1	1	2	1	1	1	1	1	1	1	1	1	1	1	1	1	1	2	2	2	2	2	2
	Gen	Mel	0.25	12	0.73	0.25	12	0.25	12	0.25	12	0.25	12	0.25	12	0.25	12	0.25	12	0.25	12	0.25	12	0.25	12
	Gen	Mel4	1	100	2	1	100	1	100	1	100	1	100	1	100	1	100	1	100	1	100	1	100	1	100
	Gen	RK758	0.5	4	1	0.5	4	0.5	4	0.5	4	0.5	4	0.5	4	0.5	4	0.5	4	0.5	4	0.5	4	0.5	4
	Cip	Mel	0.25	6	0.49	0.25	6	0.25	6	0.25	6	0.25	6	0.25	6	0.25	6	0.25	6	0.25	6	0.25	6	0.25	6
	Cip	Mel4	1	50	1.5	1	50	1	50	1	50	1	50	1	50	1	50	1	50	1	50	1	50	1	50
	Cip	RK758	0.25	2	0.5	0.25	2	0.25	2	0.25	2	0.25	2	0.25	2	0.25	2	0.25	2	0.25	2	0.25	2	0.25	2

Table S2. MIC (**µM**) determination over a 10-day consecutive passage of *Psuedomonas aeruginosa* 27853 in antimicrobial compounds and antimicrobial combinations. FIC = fractional inhibitory concentration. Gen = gentamicin. Cip = ciprofloxacin. Mel = Melimine.

			Combination MIC																						
	**Day**		**0**		**FIC**	**1**		**2**		**3**		**4**		**5**		**6**		**7**		**8**		**9**		**10**	
**P. aeruginosa 27853**	**Gen**		4		-	16		64		64		128		256		256		256		256		512		512	
	**Cip**		0.25		-	0.5		2		4		4		4		8		16		16		16		32	
	**Mel**		250		-	250		250		250		250		250		250		250		250		250		250	
	**Mel4**		500		-	500		500		500		500		500		500		500		500		500		500	
	**RK758**		100		-	100		100		100		100		100		100		100		100		100		100	
	**Gen**	**Cip**	4	0.25	2	4	0.25	4	0.25	8	0.25	8	0.25	16	0.5	16	0.5	16	0.5	16	1	16	1	16	1
	**Gen**	**Mel**	0.12	50	0.23	0.12	50	0.12	50	0.12	50	0.12	50	0.12	50	0.12	50	0.12	50	0.12	50	0.12	50	0.12	50
	**Gen**	**Mel4**	4	500	2	4	500	4	500	4	500	4	500	4	500	4	500	4	500	4	500	4	500	4	500
	**Gen**	**RK758**	1	12	0.37	4	50	4	50	4	50	4	50	4	50	4	50	4	50	4	50	4	50	4	50
	**Cip**	**Mel**	0.03	50	0.32	0.03	50	0.03	50	0.03	50	0.03	50	0.03	50	0.03	50	0.03	50	0.03	50	0.03	50	0.03	50
	**Cip**	**Mel4**	0.06	125	0.49	0.06	125	0.06	125	0.06	125	0.06	125	0.06	125	0.06	125	0.06	125	0.06	125	0.06	125	0.06	125
	**Cip**	**RK758**	0.03	25	0.37	0.03	25	0.03	25	0.03	25	0.03	25	0.03	25	0.03	25	0.03	25	0.03	25	0.03	25	0.03	25

Table S3. Minimum inhibitory concentration (MIC) determination over a 30-day consecutive passage of *S. aureus* 25923 and P. aeruginosa 27853 in sub-inhibitory concentrations of peptidomimetics.

		**MIC (µM)**			
	**Day**	**0**	**10**	**20**	**30**
S. aureus ATCC 25923	Melimine	25	25	25	25
	Mel4	100	100	100	100
	RK758	8	8	8	8
P. aeruginosa ATCC 27853	Melimine	250	250	250	250
	Mel4	500	500	500	500
	RK758	100	100	100	100
